# Digging down or scratching the surface: how patients use metaphors to describe their experiences of psychotherapy

**DOI:** 10.1186/s12888-021-03551-1

**Published:** 2021-10-27

**Authors:** A. Malkomsen, J. I. Røssberg, T. Dammen, T. Wilberg, A. Løvgren, R. Ulberg, J. Evensen

**Affiliations:** 1 Division of Mental Health and Addiction, Oslo University Hospital, P.O. box 4959, Nydalen, N-0424 Oslo, Norway; 2 University of Oslo, Institute of Clinical Medicine, P.O. box 1171, Blindern, 0318 Oslo, Norway; 3 Department of Behavioral Medicine, Faculty of Medicine, University of Oslo, Oslo, Norway; 4 Department of Psychiatry, Diakonhjemmet Hospital, Box 85 Vinderen, 0319 Oslo, Norway; 5 Nydalen Outpatient Clinic, P.O. box 4959 Nydalen, N-0424 Oslo, Norway

**Keywords:** Major depressive disorder, Cognitive behavioral therapy, Psychodynamic therapy, Metaphor

## Abstract

**Background:**

In the present study, we wanted to explore which metaphors patients suffering from major depressive disorder (MDD) use to explain their experience of being in therapy and their improvement from depression.

**Methods:**

Patients with MDD (*N* = 22) received either psychodynamic therapy (PDT) or cognitive behavioral therapy (CBT). They were interviewed with semi-structured qualitative interviews after ending therapy. The transcripts were analyzed using a method based on metaphor-led discourse analysis.

**Results:**

Metaphors were organized into three different categories concerning the process of therapy, the therapeutic relationship and of improvement from depression. Most frequent were the metaphorical concepts of surface and depth, being open and closed, chemistry, tools, improvement as a journey from darkness to light and depression as a disease or opponent.

**Conclusions:**

Patient metaphors concerning the therapeutic experience may provide clinicians and researchers valuable information about the process of therapy. Metaphors offer an opportunity for patients to communicate nuances about their therapeutic experience that are difficult to express in literal language. However, if not sufficiently explored and understood, metaphors may be misinterpreted and become a barrier for therapeutic change.

**Trial registration:**

Clinical Trial gov. Identifier: NCT03022071. Date of registration: 16/01/2017.

## Background

“My point is that illness is not a metaphor, and that the most truthful way of regarding illness—and the healthiest way of being ill—is the one most purified of, most resistant to, metaphoric thinking.” This statement is made by Susan Sontag in her book Illness as Metaphor from 1978, in which she examined the metaphorical language of AIDS and cancer, and the damaging implications of the conceptual metaphors she identified [1].

The linguists Lakoff and Johnson introduced the idea of conceptual metaphors in their book Metaphors We Live By [2]. According to the Conceptual Metaphor Theory (CMT), the meaning we ascribe to abstract concepts depends not only on the knowledge we get from culture and experience, but also on the way our abstract thought is structured in terms of concrete metaphorical concepts [3]. Since people suffering from mental illness are often wrestling with abstract, existential concepts, metaphors may act as a bridge between the abstract and the concrete. Stott et al. point out that emotion is a prototypical example of an abstract concept exceedingly difficult to express without using metaphors [4]. One example of a concrete conceptual metaphor is anger. Anger is often viewed as a hot fluid in a container inside our bodies, evidenced by expressions like “keeping a lid on” or “boiling with rage” [4]. Metaphorical concepts shape the way we perceive the world by highlighting certain aspects and hiding others, in turn affecting how we interpret situations and ultimately how we behave [2].

The use of metaphors in therapy is a topic that has been addressed in psychodynamic therapy, cognitive therapy, narrative therapy, trauma therapy and many other therapeutic approaches [5–9]. Coll-Florit et al. present a summary of the main English language studies that explore the most used conceptual metaphors describing the experience of suffering from depression [10]. They find that conceptual metaphors of darkness, burden, descent, bounded space, journey and enemy are among the most common.

Mathieson et al. have shown that metaphors are frequent in cognitive behavioral therapy (CBT), counting a total frequency of 31,5 per 1000 words of cognitive therapy sessions [11]. This study, however, only measured the frequency of metaphors, and did not explore how metaphors were used by either patients or therapists. Levitt et al. closely examined two patients’ use of the “burden metaphor” related to depression [12]. Results indicated that in the successful course of therapy the metaphor of “being burdened” had been transformed to a metaphor of “unloading the burden” over the course of therapy, which did not happen in the other, less successful course of therapy.

Sarpavaara and Koski-Jännes examined the use of metaphors in the first two sessions of a motivational interview of 21 patients suffering from substance abuse [13]. They found several conceptual metaphors, the most common being “change is a journey”, used by 12 participants, and patients who framed themselves in a positive way within this metaphor had better treatment outcomes than those with a negative framing of themselves. Although they do not claim to prove a causal relationship between the conceptual metaphor and treatment outcome, they conclude that patients often use metaphors when talking about change and that these metaphors seem to be important. That metaphorical restructuring can be effective to reduce mental stress was also found to be true in a micro-counseling scenario by Hu et al. [14].

Mathieson et al. has developed a metaphor workshop shown to improve therapists’ metaphor awareness and confidence [15]. This research group also examined whether better attention to metaphoric language by CBT therapists increased client ratings of alliance [16]. They did this by training 12 therapists to better attend to patient metaphors and bring metaphors into case conceptualizations, which resulted in a significant increase in ratings of therapeutic collaboration, session satisfaction (measured by Session Rating Scale) and a non-significant increase on the working alliance (measured by Working Alliance Inventory). By testing patients’ and therapists’ preference of figurative language they found that working metaphorically may be most effective when both the therapist and the patient enjoy speaking metaphorically.

It is important to remember that not only patients use metaphors to describe therapeutic processes – researchers and therapists are no exception. For example, Tay has presented a way of using the conceptual metaphor of “present is past” as an alternative model to interpret and explain transference in psychotherapy – thus providing an alternative and complementary way to understand how patients construct the relationship between the past and present and the way this plays out in therapy [17]. This shows the potential of metaphor analysis in the development of psychotherapeutic theory and technique. Furthermore, Stiles & Shapiro has critiqued process-outcome psychotherapy research for implicitly subscribing to a drug metaphor; a conceptual metaphor implying that therapy consists of active ingredients supplied by the therapist, with an integrity comparable to the chemical purity of drugs and presupposing a passive patient [18].

Several qualitative meta-analyses have shown that a better understanding of the therapeutic process from the patients’ perspective is important to increase the effectiveness of therapy [19–21]. Metaphors are often used to describe what is otherwise difficult to express. Kauschke et al. found that depressed patients are able to understand and produce metaphors for internal states similar to non-depressed controls, contradicting earlier assumptions that patients with depression show a concreteness bias [22]. Neuroscientific research shows that metaphors, even idiomatic expressions, engage us stronger on an emotional level than literal expressions, resulting in stronger activation of brain structures associated with processing emotional stimuli [23, 24]. These are in sum important arguments for studying which metaphors depressed patients use to explain their experience of being in therapy. This may bring a new and important perspective on how patients engage with and improve from psychotherapy. To the best of our knowledge, no other previous researchers have done this.

In this study, we aim to explore which metaphors 22 patients suffering from major depressive disorder receiving either cognitive behavioral therapy (CBT) or psychodynamic therapy (PDT) use to explain their experience. Our research question was: Which metaphors do patients use to explain their experience of being in therapy and their improvement from depression?

## Methods

### Design, ethics and data collection

The present study took place at two public psychiatric outpatient clinics in Oslo, in which patients suffering from a wide range of mental illnesses are treated. These clinics are part of the specialist health care system and require that patients are referred by a doctor, most often a general practitioner. The study is part of the ongoing Norwegian project on Mechanism of Change in Psychotherapy (MOP) [25]. The aim of MOP is to examine moderators and mediators in CBT and PDT for patients with MDD to develop a better understanding about what works for whom and how. The participants were randomized to either CBT or PDT; the CBT consisted of 16 sessions and three monthly booster sessions, and the PDT consisted of 28 sessions. Clinical assessments were conducted at baseline, during therapy, at the end of therapy and at follow-up investigations 1 and 3 years after treatment termination. Inclusion and treatment in the MOP-project is still ongoing, so outcome data for the participants are not yet available.

The Central Norway Regional Ethics Health Committee (REC South East 2016/340) approved the MOP-study, including the qualitative interviews. Informed consent was obtained from all participants.

### The interview

All patients from the initial phase of the study were invited to a qualitative in-depth interview after completing therapy. No selection criteria applied, and patients were invited to participate in the interview independent of sociodemographic factors, comorbid diagnosis or progress in therapy (factors that were all unknown to the interviewer). A few weeks after the end of therapy the second author conducted these interviews with a focus on patients’ positive and negative experiences with treatment, the therapeutic processes and therapeutic gains. The interviews lasted 45 to 60 min and took place at the outpatient clinic where the patients had received therapy.

The interviews were not designed to specifically explore the use of metaphors. In a few cases, the interviewer introduced a metaphor into the conversation. These metaphors were excluded from the study. The interviewer aimed for an informal and supportive tone, using semi-structured interviews and encouraging the participants to elaborate on themes of relevance. The patients were questioned about therapy in general, what had been helpful and not helpful, how therapy affected their relationships and to what extent they could use something from therapy in their everyday life. The interviews did not focus specifically on metaphors, meaning the interviews were not conducted in order to do an analysis of the metaphors used be the patients. Patients where, however, given time to explore and verbalize their experience of the therapeutic process. Some examples of the questions asked were: How did you experience being in therapy? What contributed to improvement in your therapy? Was there anything in therapy that you experienced as not being helpful? How did therapy influence relationships and other important aspects of your life? Is there anything from therapy that you can use today or in the future? A research assistant transcribed the interviews and anonymized all the transcriptions.

### Participants

A total of 22 participants were included in this qualitative study, 15 females and seven males. Mean age at inclusion were 26 years (range 22–48). The inclusion criteria were fulfilling the criteria of MDD according to the DSM-IV (based on a clinical interview and MINI), age 18–65 years, the ability to understand, write and speak a Scandinavian language, and willingness and ability to give informed consent [26]. Exclusion criteria were current or past neurological illness, traumatic brain injury, current alcohol and/or substance dependency disorders, psychotic disorders, bipolar disorder type 1, developmental disorders, and mental retardation. The level of depression was mean 24 (range 8–32) measured with the Hamilton depression rating scale, indicating a moderate level of depression [27]. A total of eight patients were diagnosed with a personality disorder, according to the Structured Clinical Interview for DSM-IV Axis II [28]. Of the 22 interviewed participants, five dropped out of therapy due to dissatisfaction. The remaining 17 participants completed the therapy.

### Therapists and treatment

The therapists, with the exception of one psychiatric nurse, were psychiatrists and psychologists. All therapists had a minimum of two years of training in CBT or PDT. In addition, they received one year of training on the principles of CBT or time-limited PDT before receiving patients for therapy. The principles of therapy in the CBT-group were based on the book Cognitive Therapy of Depression by Aaron Beck et al. [29]. The therapy in the PDT-group was based on the general psychodynamic principles as described in the book Long-term psychodynamic psychotherapy by Glen O. Gabbard [30]. Furthermore, the PDT-treatment was based on the short-term psychodynamic psychotherapy (STPP)-manual used in the “First Experimental Study of Transference-Interpretation” [31].

Experienced clinicians monitored adherence to the treatment principles in both therapy groups in weekly group supervisions throughout the therapy period. Video recordings from the therapy sessions were reviewed by the group with focus on the initial phase of treatment, case formulation, individual treatment strategies and termination of therapy. Few other qualitative studies of this kind run a similarly strict fidelity control.

### Analysis

The object of our study was to explore which metaphors patients used to describe their experience of being in therapy and their improvement from depression. We analyzed the transcripts using a method based on the metaphor-led discourse analysis presented by Cameron et al. [32]. We operationalized the analysis using a 4-step procedure, based on the method used by Mathieson et al. [11].

First, the first author read through all interviews to familiarize himself with the data. Second, the first and last author worked through the data looking for all possible metaphors and collected them in a separate document. Third, each metaphor was analyzed to check if it met the required criteria of metaphors. Our definition of metaphor is “a figure of speech that implies a comparison between two unlike entities” – a broad definition that serves our purpose [4]. Lastly, the metaphors were coded as metaphorical when there was a contrast or incongruity between the meaning in the context and a more literal meaning [11, 33]. When all metaphors had been identified and collected by the first author, all authors read the collection of metaphors and gave feedback. Based on discussion of the material, we grouped the metaphors into several categories. The authors have different therapeutic orientations: J.E., T.D. and J.I.R. are CBT-therapists, T.W. and R.U. are PDT clinicians. A.L. and A.M. have no specific therapeutic orientation. This is made transparent in accordance with the checklist of reporting qualitative research by Tong et al. [34] .

## Results

Our analysis resulted in the identification of several metaphors used by patients to make sense of their therapeutic experience. We organized the metaphors into three different categories concerning 1) the therapeutic process 2) the therapeutic relationship 3) the experience of improvement from depression. All categories of metaphors are shown in Table 1.

**Table 1:** Conceptual metaphors used by patients to describe their experience of therapy and improvement from depression

Category of Metaphors	Conceptual Metaphors	Examples of metaphors
**The therapeutic process**	Surface and depth ^2^	Digging down^3^, getting to the root, removing dental stones.
	Tools^2^	Get tools^2^, build myself up.
	Sorting and organizing^3^	Picking up pieces, finding a missing piece of the puzzle, untangle threads^3^.
	Cleaning and emptying^3^	Sweep the dirt, emptying the garbage, clean up, ventilate.
**The therapeutic relationship**	Openness^2^	Opening up^3^, being closed.
	Chemistry^3^	Good chemistry^3^.
	Temperature^3^	Cold relationship, cold therapist, warm therapist^3^.
**Improvement from depression**	Disease^3^	The disease talking^3^, remove the megaphone.
	Opponent^3^	Monsters inside me, a saboteur.
	Stuck and loosened^3^	Something loosened^3^, being stuck, oiled the machinery.
	Up and down^3^	Reduce the fall, raising the floor.
	Darkness and light^2^	Everything is dark^3^, a spring morning.

Metaphors used by all, most and some patients are numbered 1, 2 and 3 accordingly. If only one patient used the metaphor, there is no annotation

As suggested by Hill et al. we indicate the recurrence and representativeness of patients’ experiences by using the labels general, typical and variant [35]. When something is mentioned by all or all but one patient it is labeled as general, in the text referred to as all patients. A metaphor is considered typical when it is mentioned by more than half the cases, in the text referred to as most patients. We use the expression some patients when the metaphor is found to be a variant represented by less than half but more than two cases. The abbreviations CBT and PDT will be used to specify which therapy the patient received. When, for example, the term “some patients (PDT)” is used, this means less than half but more than two cases in the PDT-group. When no abbreviation is used, it means that all patients in both groups are included. All patient metaphors are written in italic.

### The therapeutic process

The patients conceptualized their experience of the therapeutic process in many different ways. The main metaphors we discovered were: 1) metaphors of surface and depth 2) metaphors of tools 3) metaphors of sorting and organizing 4) metaphors of cleaning and emptying, as presented in Table 1.

#### Metaphors of surface and depth

Most patients explained their therapeutic process using metaphors of surface and depth. Some patients (PDT) used verbs like “diving into”, “drilling down” and “digging down” about how they experienced their conversations with their therapist. One patient (PDT) said that therapy helped her by “dragging” what she had “suppressed” (painful emotions) “up and into the light”. Several of the patients in the PDT-group expressed that they got a deeper understanding of themselves and others during therapy. One patient (PDT) said that therapy gave her “more insight into myself and some deeper understanding”. Another patient (PDT) explained it like this: “I actually think she recreated some of my relational difficulties during therapy, and that has made me more mindful of … more aware on a deeper level than I was before.”

Some patients (CBT) who expressed general dissatisfaction with their therapy, often felt that they “didn’t get deep enough” or that the therapy only “scratched the surface”. One patient (CBT) explained the difference between surface and depth in a characteristic way: “I felt that we never got to a point where ‘Listen, now we are really going to go into the depth here.’ I think that when positive thinking and exercise doesn’t help, there must be something deeper. I really believe in getting to the root of the problem, that things lie hidden more in the unconscious, in childhood, and in the past.” Another patient (CBT) also thought that they did not go “deep enough” because “it just didn’t hurt enough” and that “it was more like going to the dentist to remove dental stones”.

Not all patients in the CBT-group expressed a need to go deep and talk about their past. One patient (CBT) even said the opposite: “The therapist wanted to talk about my past to find out why I had become the way I am. And I think that’s kind of unnecessary because you can’t do anything about the past anyway.” Another patient (CBT) felt that working with the present issues solved his past problems as well: “This was CBT, so I guess it revolves more around what you’re doing right now, in everyday life and how I’m feeling right now. And if I have a feeling that this relates to something way back in time, then I guess it resolves those things too.”

One patient (PDT), who had tried CBT a few years back, explained her experience like this: “Maybe the cognitive (therapy) raised me up faster than the psychodynamic, but I went down again pretty fast and got the same problems. ( …) But this is a disease (depression) that maybe goes deeper, therefore it takes longer time.” The patient goes on to explain the same idea with yet another metaphor: “It’s a bit like when you eat something with a lot of sugar; you get lots of energy, but you also go down really fast. But if you eat a slice of whole grain bread, then you keep stable for a longer time. I think it’s the same here … both can help, but I think the psychodynamic one goes deeper.”

#### Metaphors of tools

Most patients used the same metaphor when asked what they expected and wanted from therapy: the tool-metaphor. One patient (CBT) said “I need to come here and get some tools” and was dissatisfied because she felt that she only got one or two useful tools during therapy. She defined a tool as a concrete advice on what to do and how to do it; like scheduling worry time to reduce the time spent worrying during the day. Another patient (PDT), who said she got “many tools” in therapy, defined a tool like this: “It’s what we have worked with, accepting myself, and asking the why-question, as she (the therapist) did, but asking it myself.” Another patient (CBT) was often angry at her children, but said she got “tools” in therapy on how to regulate her anger using cognitive reframing techniques. One patient (CBT) built upon the same metaphor of tools by the following statement about her therapy: “She (the therapist) continued to give me tools I could use to build myself up again more and more.”

#### Metaphors of sorting and organizing

Some patients viewed therapy as a process of sorting and organizing their thoughts. One patient (PDT) said: “It’s like a jigsaw puzzle. When you begin (therapy) you just have a couple of pieces and some corners, and you start by putting down the edges. After a while, it’s almost as if your unconscious keeps assembling pieces.” The patient explained that she sometimes wanted to give up because she could not find more pieces for her puzzle and her jigsaw pieces were “scattered all over the place”, but then “my therapist would then give me a new piece, like ´this is the one you’re missing´.” Another patient (PDT) also used the metaphor of therapy as a jigsaw puzzle, but said that the therapist was the one assembling the pieces: “He (the therapist) kept some kind of timeline, I guess he assembled a few pieces of the puzzle himself.” Some patients expressed the same idea, but did not use the same metaphor. Instead they used terms like “sorting out” and “organizing” their thoughts.

A few patients used another metaphor for explaining how the therapy had helped them sort out their thoughts: the metaphor of threads. A few patients used the metaphorical concept of a ball of threads to emphasize how they “untangled” their own thoughts with help from the therapist. One patient (CBT) said: “If she (the therapist) said ‘okay, what makes you say that?’ then I could start unwinding, and say ‘because this and that’.” (…) And then you see the strings holding the dolls inside your head.” One patient (PDT) put it like this: “I started to understand things a bit better, like, I started to untangle some parts and tying up the loose ends.” Another patient (PDT) said it was helpful to find some “common threads” because, as she said: “Sometimes you need to pull some red threads from your past to find out how things that have happened earlier indirectly influence the way you are today.”

#### Metaphors of cleaning and emptying

A few patients used metaphors that expressed a sense of cleaning or catharsis to explain the process of therapy. One patient (PDT) said it was a relief just to express her distress to someone who would listen, to “sweep” her “dirt” over to the therapist every week, and also used the metaphor of song: “Every week I get some confirmation, I get help. Every week she (the therapist) is there to listen to my song of complaint.” Another patient (PDT) conceptualized therapy in the same way, but used a different metaphor: “I think that emptying the garbage once a week … it feels pretty nice. ( …) After I got home I was pretty tired and felt really empty. ( …) But I often felt a bit lighter, like, okay, I have cleaned up a bit.” To ventilate is also an important part of cleaning, and one patient (CBT) used this metaphor when she spoke about her therapy: “It felt good just to ventilate my thoughts with someone. I often looked forward to … to just ventilate.”

### The therapeutic relationship: openness, chemistry and temperature

Most patients used the metaphor of opening and closing to describe their therapeutic process and their relationship to the therapist. Some patients felt it was scary and difficult to be “open” at the start of therapy. One patient (CBT), who categorized herself as a “closed person”, said that “opening up” was an important part of her initial improvement process. Another patient (PDT) said the same: “In the beginning it was a bit difficult to open up to an unknown person, especially since I find it difficult to talk about feelings and such to those closest to me. ( …) After a while I opened up more, and told things I didn’t really want to tell anyone.” One patient (CBT) emphasized the importance of opening up to the therapist: “It’s important to open up completely ( …) Because if I don’t tell everything from A to Z then it’s really difficult for the therapist.” Openness was not only regarded as important by patients within therapy, it was also regarded as a goal in itself. When asked how she experienced improvement in therapy, one patient (CBT) said that “I’m more open”. Another patient (CBT) said that openness in therapy made it easier to “open up” to his wife. When asked what was the most important result of therapy, one patient (PDT) combined the concept of openness with a spatial metaphor: “That I got room to open up.”

For a few patients, it was important that the therapist was open as well. When asked what characterized an open therapist, one patient (CBT) said: “Quite calm … eye contact … that she keeps asking follow-up-questions.” Another patient (PDT), who found her therapist to be “too closed” and “very serious”, wished that he had been “More open. Not those long silences. That he asked more. Kept the conversation going.”

Some patients used the metaphor of chemistry to explain their relationship with the therapist. One patient (PDT) made the connection between the chemistry and her ability to be open in therapy, claiming she got better results with a previous therapist she had “good chemistry” with because she “could open up more”. One patient (PDT) believes that chemistry in therapy is about how “similar” the patient and therapist are, especially concerning values. Another (CBT) said that “good chemistry” is more a matter of “mutual respect”.

A final metaphor some patients use to describe their therapist was the metaphor of temperature. One patient (PDT) described her therapeutic relationship in the following way: “I had the feeling it was kind of cold. ( …) It was like a wall. ( …) There was a distance there. ( …) It was like we were on two completely different planets” Another (CBT) said that: “If I had to describe her (the therapist) with one word, I would say she was warm”. One patient (CBT), who didn’t get a good relationship with his therapist, said that “I didn’t feel any warmth” and wished that his therapist had provided him with coffee. He explained that such a small act of kindness would help him by “helping me to lower my guard, enabling me to feel instead of just analyzing”.

### Improvement from depression: disease, opponent, stuck and darkness

When trying to explain their process of improvement, the patients used many different metaphors. We will highlight three metaphors concerning improvement: depression as a disease and opponent, a sense that something previously stuck is loosened, and improvement understood in terms of darkness and light.

Some patients made a distinction between themselves and “the disease”, referring to the depression. This seemed to relieve some patients of guilt, as explained by one of the patients (PDT): “It’s about realizing that this is a disease, so in a way, it’s not my fault.” Another patient (CBT) said he wanted his therapist to “arrest him” when he spoke in a depressive manner, and to say to him that “This is your disease talking, you have to stop letting it take over”. One patient (PDT) said she experienced improvement when the depression lost its control over her: “It was like the monsters living inside me didn’t have as much authority anymore”. This framing of depression as an opponent was also used by another patient (PDT) who said she carried inside her “a saboteur who always ruins things for me”. Another patient (CBT) said that therapy helped her “remove the megaphone” from her destructive thoughts so that she could hear her other thoughts as well.

A few patients used the metaphor that “something loosened inside” or “something clicked inside” about their improvement from depression. One patient (PDT) said that “It’s difficult to explain, but it felt like … something loosened”, another patient (PDT) explained that “In a way it’s just like it just suddenly loosens.”

The metaphor of darkness seemed to be a common way of framing the depression, and expressions like “go into these darks rooms”, “I’ve got this darkness”, “everything is dark” and “dark patterns of thought” were common. A few patients described improvement as appearance of light, shown in expressions like “it’s much lighter, like a spring morning” and “people say I’m shining”.

Most patients used metaphors to describe their improvement from using medication (antidepressants, SSRI). One patient (PDT) said: “I started noticing that things were easier, it was like someone had oiled my machinery” and another (PDT) that medication “took the edge off”. One patient (PDT) said that the medication made him “flat”, but that it stopped the “waves of sadness” from becoming overwhelming. Some patients used the concept of verticality to explain how they experienced that medication reduced the risk of falling back down into depression. One patient (CBT) said that when he started taking medication (SSRI) it was like “now I’m finally in that safe … that safe haven” because the medication “raise the floor, it’s like, you don’t fall such a long way down”. That medication reduced “the fall” was also supported by a patient (CBT) who said that the medication (SSRI) helped by not letting her “go down into the dark”.

To explain how they felt after therapy, patients used several different metaphors. One patient (PDT) used an aerial metaphor: “I feel like I’m an airplane on the runway. And I hope it takes off. I don’t know if it will take off, but I feel I’m well equipped now”. The same patient used a botanical metaphor to describe her improvement: “She (the therapist) planted some seeds. Even when I didn’t want to admit it, there was a seed that started to grow inside me. ( …) If you imagine that it’s weed all over the garden, it was like ( …) she planted a rose there.” One patient (PDT) used a digital metaphor and said that he felt “rebooted” after therapy, another (PDT) said that therapy gave him “more weight” so that he could “sail a bit steadier and safer in the storm.”

## Discussion

This study aimed to explore which metaphors patients use to describe their experience of therapy and their improvement from depression. Our results show that patients use many different metaphors, but that there are some conceptual metaphors that are used by most patients. We will now discuss these metaphors individually.

### Surface and depth: what patients need to explore in therapy

In an article about the metaphor of depth and the ways in which it can mislead, Wachtel makes the point that we are sometimes taken prisoners by our therapeutic metaphors [36]. The metaphor of surface and depth may be considered a conceptual metaphor. This metaphor has become so common and compelling that one may tend to forget that it is a metaphor, and thereby forget that there can be alternative ways of conceptualizing therapy.

We find that most patients believe it is important to go deep in therapy. At the same time, there seems to be less consensus on what it really means to go deep. While some patients think that depth equals painful emotions, others believe you go deep by talking about past experiences or by exploring unconscious processes.

It seems that many patients believe that the exploration of present issues in CBT is “shallow”, and that they only scratch the surface of their problems in therapy – even though they improve. Many patients were dissatisfied because they felt they hadn’t dug deep enough into the depth of their psyche. Our impression is supported by a qualitative comparison of CBT and PDT by Nilsson et al. [37]. They found that the statement “getting to the root of things” was used by 73% of the satisfied PDT-patients, while none of the satisfied CBT patients used this metaphor. The same was pointed out by De Smet et al. in a qualitative analysis of depressed patients receiving CBT and PDT, where they found that many patients think that CBT is too superficial [38]. This is a critique of CBT that we believe is of great clinical importance, especially because it may be avoided by exploring the patient’s metaphorical understanding of therapy.

It seems vital to step into the patient’s conceptual metaphors of therapy to understand what surface and depth means to the patient. What does the patient mean when she says that the therapy is not deep enough? Does the patient think that something important has been avoided, or overlooked? Has the emphasis on present issues, which is a crucial part of CBT, been conceptualized as “scratching the surface” by the patient? Or has the therapy been too cognitive and with too little emphasis on painful emotions? These are just some interpretations based on our results. This is important because even though the therapy is effective, our results indicate that many patients are still not satisfied if their wish to “go deep” has not been sufficiently met. Therapists may take time to explain the theoretical rationale and the conceptual metaphors behind their therapeutic approach when starting therapy, or whenever needed. It may also help therapists to broaden their horizon by studying alternative metaphors. Some alternative metaphors, like horizontal depth instead of vertical depth, or going from depth to breadth, are discussed in an article by Wachtel [36].

### Tools: what patients need to improve

We identified that patients in both groups had the same expectation of therapy and used the same metaphor describing it: to get a mental tool to solve their problems. This may or may not be possible and feasible, depending on the definition of a “tool”. It seems to us that many patients are unsure of what really constitutes a mental tool; all they know is that they want one. We think it may be important that the therapist engages in this metaphor in the same way as mentioned above. Does the patient want gardening tools to clean her mental weeds and fertilize the soil of her soul? A mental wrench or screwdriver to tighten or loosen parts of her mind? A cerebral knife to cut some of her thoughts out of her mind? Or does she dream of a psychological multitool that does all of the above?

In two previous articles on the same patient material we found that both patients who had received CBT and PDT wanted tools to help them out of the depression [39, 40]. By exploring the metaphor of tools more closely when it is used by the patient, the therapist may get a better understanding of what the patient actually needs. In addition, by being asked to elaborate the metaphor, the patient may increase her own understanding of what she is actually seeking in therapy when she is asking for a tool. If the therapist manages to explore this metaphor with the patient and re-imagine her metaphorical concept of a tool so that it fits with the therapy, we think the patient satisfaction will increase.

### Openness, chemistry and temperature: what patients need from the therapist

Openness, chemistry and warmth seem to be the most important metaphors concerning the therapeutic relationship. It may not be obvious at first that these terms are in fact metaphors because they may represent what are often labeled as “dead metaphors”. Dead metaphors are particular words or phrases that have become linguistically attached to a particular meaning [4]. They may be dead, but this does not mean they should go unrecognized. As suggested by Witztum & van der Hart, dead metaphors may be brought back to life and become excellent points of departure for therapy [41].

As an example, imagine that the patient says to the therapist that she is struggling to “open up” in therapy. How can the therapist unwrap and re-awaken this metaphor? To unwrap its literal meaning, the therapist may ask questions about what this metaphor really means to the patient: it may mean she is holding back particular parts of her story; it may mean that she is not being honest about what she is feeling; or it may mean that she is not showing her true feelings. To re-awaken the metaphor, the therapist may need to use the same metaphorical concept and try to make sense of how this metaphor of openness affects the patient. Does it feel vulnerable to be open because she associates openness with an open door – where a burglar may sneak in – or does openness make her afraid like the open door of a lion’s cage or the way an open door of the freezer will make the ice melt and destroy the food inside? The point of “stepping into” the patient’s own metaphor in this matter is ultimately to build a cognitive bridge and to change the way the patient thinks and feels within the metaphoric realm [4]. Working with metaphors may make it easier for therapists to understand the patient’s experience (of existing, being depressed, being in therapy). In turn, this may give the therapist a better understanding of what the patient needs, creating more empathy and strengthening the working alliance. The importance of a discovery-oriented, collaborative style of metaphorical elaboration is shown in a study by Angus and Rennie [42]. In an in-depth analysis of four dyads they found that a mutually shared understanding of metaphors was important to avoid misunderstandings. They found that a shared understanding could be achieved by attentive listening strategies that encouraged patients to present their particular associations to a metaphor.

Most patients value openness in therapy, but it seems that many patients find openness in the therapeutic setting to be difficult, especially if they feel a lack of chemistry with the therapist. Landau et al. studied the fear of exposing oneself by exploring the common conceptual metaphor of a “true self” as a physical entity that must be protected from external threats [43]. While they do not discuss openness specifically, it would make sense that being open will expose this “true self” to danger, which may partly explain the patients’ reluctance.

Some patients in our material used yet another metaphorical concept to explain why they hesitated to open up: the temperature of the therapist. One patient said he did not feel any warmth from the therapist and suggested it would have helped if the therapist had offered him a cup of coffee. Interestingly, Williams and Bargh have found that experiencing physical warmth – e.g. by holding a warm cup of coffee – promotes interpersonal warmth [44].

### Improving from depression: darkness and light

We find that our patients use the same conceptual metaphors that are found by other researchers in other countries [10]. This supports the idea that conceptual metaphors can cross barriers of culture and language. For example, the conceptual metaphor of light and darkness as a way of experiencing depression is not unique for our patients – it is part of our culture. A metaphorical analysis by Forceville of nine short, wordless animation movies concluded that the films featured two dominant metaphors: depression as dark monster and depression as a dark confining space [45]. The metaphors of darkness and light are also found by El Refaie in two graphic memoirs, and Schoeneman et al. show that this conceptual metaphor of depression as a struggle in darkness is by no means new, as it is also found – for example – in the The book of Job [46, 47].

While the connection between depression and darkness seem to be manifest in western culture, one could wonder whether the connection between depression and darkness is universal. Interestingly, the same metaphors of depression as darkness and being confined in a dark space was also found to be dominating in the Iranian society [48]. In a study by Schwartz et al. they found that people who prefer darkness to light are more prone to negative emotional experiences and symptoms [49]. It also seems that darkness and light actually have a biological influence on depression, although the mechanisms are still largely unknown [50, 51].

Conceptualizing depression as being confined in a tight space, way down, alone in a void-like darkness has the potential to enhance the patient’s feeling of being isolated, abandoned and exposed to horror. Exploring such metaphors may however clarify, expand and validate the patient’s experience. It may capture the patient’s current feelings, but it may need to be counterbalanced by the therapist’s metaphors – perhaps metaphors of expansion, elevation and light.

### Improving from depression: depression as opponent or disease

A few patients seemed to think and feel their depression had its own unique character that somehow had occupied their body and/or mind. Some even said that the depression had its own voice that they could argue with or force to silence. The metaphorical concept of the depression as an opponent and a disease is not a new one, and has gained popularity over the years, as found by a multinational Latin American study by Reali et al. [52]. Susan Sontag emphasized the negative effects of looking at a disease as an opponent in her book, a point that has later been followed up by others later [1, 4, 53]. Heide calls the opponent-metaphor “the agonistic metaphor” and points out that while the metaphor may motivate patients to “fight their depression”, it also has several potential negative consequences, like making the patient more hostile to herself and her own thoughts and feeling. It has also been shown that trying to suppress and remove thoughts and feelings, as one would try to do in a battle, often is counterproductive [53]. Whether a biogenetic disease-framing of depression is helpful is an empirical question, and in a quantitative synthesis by Kvaale et al. they found that biogenetic explanations for mental disorders are negatively associated with blame, but positively associated with perceived dangerousness (for schizophrenia) and with desire for distance [54]. Reali et al. found that people who conceptualized depression as a place-in-space (e.g. “a dark place”) favored social-related causal explanations, while the opposite was true when depression was framed as a disease, indicating that metaphorical framing of depression as a disease may also affect the way patients look for solutions [52].

When patients frame their depression as a disease, and this framing make them believe that nothing can be done except using medication, it may be wise to explore what kind of physical disease might be the best analogy for their depression [4]. If a patient views her depression as a viral infection – comparable to the flu – it may be tempting to stay in bed and wait for it to pass, an approach seldom effective in the treatment of depression. Rather, if viewed as a systemic disease of multifactorial etiology – comparable to type 2 diabetes – the analogy might stimulate exercise, a healthy diet and an active life – in addition to the use of medication.

Another challenge when dealing with patients who are using medication as well as receiving psychotherapy is that this combination may send out a mixed message. The use of medication sends out the message that this is a physical disease, requiring pharmacological treatment. At the same time, the therapist communicates that the problem is psychological by offering psychotherapy. Combining medication and psychotherapy is shown to be more effective than pharmacotherapy alone [55], so this mixed message should not stop therapists from suggesting this combination. Stott et al. provide therapists with a possible solution to the problem in the form of a metaphor: medication as training wheels [4]. Using this metaphor, medication may help to reduce symptoms and thereby make it easier for patients to engage in therapy. Nonetheless, it is impossible to learn how to ride a bike simply by attaching training wheels. The aim of psychotherapy might be to learn the patient how to ride – or live – so that they may be less reliant on training wheels – or medication – in the future.

### Strengths and limitations

Interviews were performed without questioning the patients specifically about their use of metaphors, as we did not plan to investigate the use of metaphors at the time interviews were conducted. The idea for this article came later, when we recognized the patients’ extensive use of metaphors during the interviews and the importance of what was being said through their use of metaphors. As the interview was not specifically designed to explore metaphors, a few common metaphors were introduced by the interviewer during the interview. These metaphors have been excluded from the analyzed material. As these occurred in small numbers and were of a common nature, their exclusion should not impact the results. The fact that the interviewer did not focus on metaphors gives us a great opportunity to analyze the use of metaphors in a “natural” conversation.

This study was not designed to differentiate the use of metaphors between patients who got PDT and CBT. We can therefore only speculate on how the two groups differ. Interestingly, we cannot find any major differences between the two groups concerning which metaphors they use to describe their therapy or improvement. Neither did we find clear differences between the groups in the way they interpret and use these metaphors. There is not much research on how the therapists in CBT and PDT differ regarding their use of metaphors. Thus, it is difficult to say how the therapeutic approach impacts the patients’ conceptual metaphors. Further research is needed to answer this question.

None of the authors have any formal linguistic training. To increase reliability, the first and last author read the transcripts independently and agreed on the identification of metaphors. We believe this method served our purpose for this article. It is a considerable strength for the analysis that the authors have such varied therapeutic orientations, as this decreases the risk of bias.

Obviously, these results do not apply directly to other patients. We suspect that additional caution of generalization is advised when studying metaphors. Nonetheless, as we read the current research, we are struck by the metaphorical similarities across different cultures and languages.

## Conclusions

We have identified several metaphors patients use to explain their experience of being in therapy, the therapeutic relationship and of improving from depression. The metaphorical concepts of surface and depth, being open or closed, tools, chemistry, light-darkness and depression as a disease or opponent have been discussed in detail. Our study is a reminder that listening closely to the patients’ metaphorical expressions can reveal lived experiences of great importance and shed light on what patients want from therapy and how they experience the therapeutic process.

## Data Availability

The data is available from the corresponding author on reasonable request.

## Abbreviations

CMT: Conceptual Metaphor Theory
BDI: Becks Depression Inventory
CBT: Cognitive Behaviour therapy
DSM-V: Diagnostic and Statistical Manual of Mental Disorders - version IV
M.I.N.I.6.0.0.: Mini International Neuropsychiatric Interview
MDD: Major Depressive Disorder
MOP: Mechanisms of change in Psychotherapy
STPP: Short Term Psychodynamic/psychoanalytic psychotherapy
